# pH-TriggeredRelease of Cinnamon Essential Oil from Sodium Alginate-Shellac Nanoparticles: Rational Design, Enhanced Stability and Antibacterial Efficacy

**DOI:** 10.3390/foods15071237

**Published:** 2026-04-04

**Authors:** Sijing Liang, Ouyang Zheng, Jing Xie, Shucheng Liu, Qinxiu Sun

**Affiliations:** 1 College of Food Science and Technology, Guangdong Ocean University, Guangdong Provincial Key Laboratory of Aquatic Product Processing and Safety, Guangdong Province Engineering Laboratory for Marine Biological Products, Guangdong Provincial Engineering Technology Research Center of Seafood, Key Laboratory of Advanced Processing of Aquatic Product of Guangdong Higher Education Institution, Zhanjiang 524088, China; liangsijing0912@163.com (S.L.); zhengouyang07@163.com (O.Z.); lsc771017@163.com (S.L.); 2 Collaborative Innovation Center of Seafood Deep Processing, Dalian Polytechnic University, Dalian 116034, China; 3 College of Food Science and Technology, Shanghai Ocean University, Shanghai 201306, China; jxie@shou.edu.cn

**Keywords:** shellac, polyelectrolytes, cinnamon essential oil, nanoparticles, pH-responsive release

## Abstract

Sodium alginate (SA)-modified shellac nanoparticles were developed as pH-responsive carriers for cinnamon essential oil (CEO) encapsulation in aquatic product preservation. Three polyelectrolytes (SA, chitosan (CS), gelatin (Gel)) were evaluated at concentrations of 0.025–0.3% (*w*/*v*). Under pH conditions simulating spoilage (6.0–7.5), SA-SNPs exhibited superior stability with minimal changes in particle size, PDI, and zeta potential, while CS and Gel systems aggregated near their pKa values. At 0.1% SA, CEO-loaded nanoparticles (SA-SCNPs) showed excellent properties: small size (160 nm), high encapsulation efficiency (90%), and pH-triggered release (77.76% at pH 7.0 via Ritger–Peppas kinetics, *n* = 0.58). FT-IR confirmed ionic and hydrogen bonding between the SA and shellac. SA-SCNPs enhanced antibacterial efficacy against *Shewanella putrefaciens* and *Pseudomonas fluorescens* and maintained stability under ionic strength (300 mmol/L NaCl) and temperature variations (−18 °C to 25 °C), attributed to SA’s cryo-resistance and steric effects. This system offers a smart delivery platform for aquatic preservation.

## 1. Introduction

Aquatic products are highly perishable, with global post-harvest losses estimated at 10–15% [1], primarily due to microbial spoilage. This results in significant economic losses and food waste. The application of natural antimicrobial agents, like cinnamon essential oil (CEO), represents a promising approach to prolong the shelf life of such perishable goods by targeting key spoilage organisms including *Shewanella putrefaciens* and *Pseudomonas fluorescens*, which are known to dominate the microbiota of spoiled aquatic products [2,3,4]. However, the practical use of CEO is limited by its volatility, poor water solubility, and susceptibility to degradation [5]. Encapsulation serves as an effective means to address these challenges by entrapping the active component within a protective carrier, thus shielding it from environmental factors and improving its bioavailability [6].

Among various encapsulation carriers, nanoparticles are particularly advantageous due to their high specific surface area and enhanced permeability [7,8]. Nevertheless, conventional nanocarriers often suffer from non-targeted release profiles, such as premature burst release, which are not aligned with the dynamic process of food spoilage [9]. To address this, intelligent pH-responsive delivery systems have been developed. Shellac, a natural polymer with a pKa of 5.6–7.0, is well-suited for such systems, as its pH responsiveness coincides with the pH increase typically observed during fish spoilage [10,11,12,13].

Despite its potential, the practical application of shellac nanoparticles (SNPs) is hindered by their inherent instability due to molecular aging [14]. Surface modification using polyelectrolytes offers a viable approach to address this challenge. Existing strategies have primarily focused on improving colloidal stability under fixed pH conditions. For instance, sodium caseinate coatings enhance the acid resistance, salt tolerance, and redispersibility of SNPs [15]; xanthan gum or sodium caseinate complexes are used to prevent SNPs aggregation under low pH conditions [16,17]. However, the spoilage of aquatic products is accompanied by a continuous increase in pH. Optimization strategies targeting fixed pH conditions cannot adapt to this dynamic change, resulting in a mismatch between carrier performance and the actual spoilage kinetics. Existing studies have neither investigated the evolution of interfacial structures under dynamic pH conditions, nor systematically explored how polyelectrolyte type (anionic, cationic, or amphoteric) and concentration cooperatively regulate carrier stability and release behavior throughout the entire pH transition—where the type dictates the interfacial stabilization mechanism and the concentration governs the compactness of the interfacial layer, both jointly determining the fate of the carrier during continuous pH variation. The absence of such systematic comparisons confines material selection to empirical approaches, ultimately leading to a mismatch between carrier performance and the dynamic process, which severely limits the practical application of such intelligent delivery systems.

Therefore, this study establishes a framework from mechanistic understanding to rational design. Through the systematic screening of sodium alginate (SA), chitosan (CS), and gelatin (Gel) at modification concentrations ranging from 0.025% to 0.3%, a library of polyelectrolyte-modified SNPs with distinct physicochemical properties was constructed. This enabled the rational selection of SA as the optimal stabilizer to produce SA-SNPs. Subsequently, by loading SA-SNPs with increasing concentrations (2%, 4%, and 8%) of CEO, the stability, release kinetics, and antibacterial efficacy were comprehensively optimized, leading to the construction of a high-performance, pH-responsive delivery system termed SA-SCNPs. This work provides a complete solution strategy and a material platform, spanning from molecular mechanisms to carrier design, for the development of intelligent preservation technologies targeting aquatic product spoilage.

## 2. Materials and Methods

### 2.1. Materials and Reagents

Cinnamon essential oil (99% purity) was purchased fromJiangxi Cedar Natural Medicinal Oil Co., Ltd. (Shanghai, China),Food grade shellac was obtained fromShanghai Yuanye Bio-Technology Co., Ltd.(Shanghai, China), sodium alginate (90% purity), chitosan (degree of deacetylation ≥ 90%, MW—100,000), gelatin (biotechnology grade), and Tween-80 (biotechnology grade) were all supplied byShanghai Macklin Biochemical Co., Ltd. (Shanghai, China), Nutrient broth and *Pseudomonas* CFC selective medium base was sourced fromBeijing Land Bridge Technology Co., Ltd. (Beijing, China). Iron agar was purchased fromQingdao High-Tech Industrial Park Haibo Biotechnology Co., Ltd. (Qingdao, China). All chemical reagent used were of analytical grade.

### 2.2. Experimental Design

**Figure 1:**
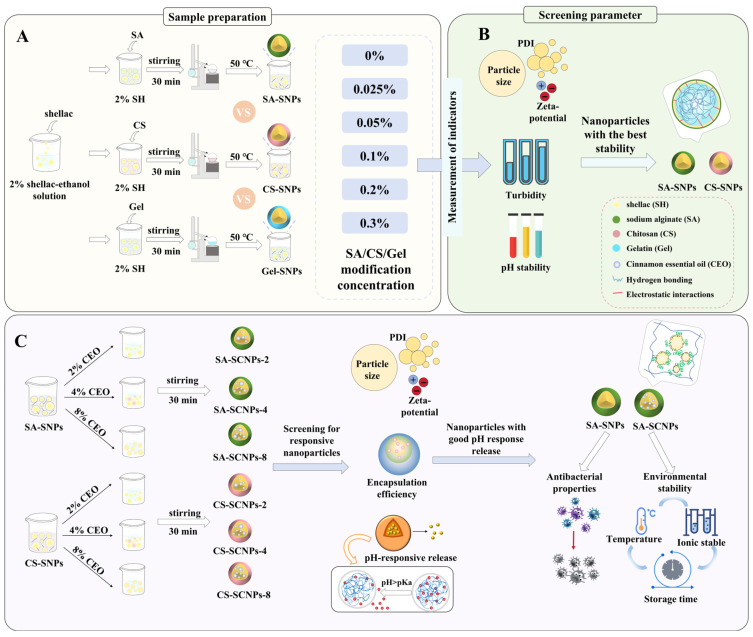
Schematic diagram of the experimental design. (**A**) preparation process of polyelectrolyte-modified shellac nanoparticles; (**B**) screening and characterization of polyelectrolyte-modified shellac nanoparticles; (**C**) preparation and characterization of cinnamon essential oil-loaded shellac nanoparticles.

The experimental design of this study is illustrated in Figure 1 and consists of two parts. First, three polyelectrolytes (Figure 1A)—SA, Gel, and CS—were selected to modify shellac nanoparticles (SNPs), preparing SA-SNPs, Gel-SNPs, and CS-SNPs with different modification concentrations (0.025%, 0.05%, 0.1%, 0.2%, 0.3%). The influence of polyelectrolyte type and concentration on SNP stability was determined through comprehensive characterization of particle size, PDI, zeta potential, and turbidity (Figure 1B). Simultaneously, based on the pH variation range during the storage of aquatic products, the influence of pH changes (6.0, 6.5, 7.0, 7.5) on the stability of the nanoparticle complexes was systematically investigated. Through comprehensive analysis, SA with a modification concentration of 0.1% and CS with a modification concentration of 0.1% were selected for subsequent experiments.

Based on the preliminary screening (Figure 1C), SA-SNPs and CS-SNPs were used as carriers to encapsulate CEO at three different concentrations (2%, 4%, 8%), forming six experimental groups labeled as SA-SCNPs-2, SA-SCNPs-4, SA-SCNPs-8, CS-SCNPs-2, CS-SCNPs-4, and CS-SCNPs-8, respectively. Corresponding unmodified CEO-loaded SNPs at the same concentrations were set as the control groups, labeled SCNPs-2, SCNPs-4, and SCNPs-8, to compare the effects of SA/CS modification on nanoparticle stability.

The pH-triggered release characteristics of the nanoparticles were examined. Following physicochemical characterization, including measurements of hydrodynamic diameter, zeta potential, encapsulation efficiency, and FT-IR spectra, the cumulative release of CEO was quantified in phosphate buffer solutions at pH 6.2, 6.4, 6.6, 6.8, and 7.0 to simulate spoilage conditions. SA-SCNPs, which showed the most desirable pH-responsive release, were selected for subsequent stability assessments, including storage stability (over 28 days), ionic strength stability (25–300 mmol/L NaCl), and thermal stability (−18 °C, 4 °C, 25 °C). In addition, the antimicrobial performance of the nanoparticles was investigated against representative spoilage bacteria, including *Pseudomonas fluorescens* and *Shewanella putrefaciens*.

### 2.3. Preparation of Polyelectrolyte-Modified Shellac Nanoparticles

Shellac (2 g) was completely dissolved in 100 mL of anhydrous ethanol by magnetic stirring (HH-J4, Changzhou Aohua Instrument Co., Ltd., Changzhou, China) at 600 rpm and 25 °C for 6 h. The obtained 2% (*w*/*v*) shellac-ethanol solution was passed through a 0.45 μm membrane filter to eliminate any insoluble matter. SA and Gel were separately dissolved in ultrapure water, yielding solutions with concentrations of 0.025%, 0.05%, 0.1%, 0.2%, and 0.3% (*w*/*v*). CS was dissolved in 1% (*v*/*v*) acetic acid solution following the same concentration gradient. Polyelectrolyte-modified SNPs were prepared using an antisolvent precipitation approach adapted from Li et al. [18]. Briefly, 12 mL of the 2% (*w*/*v*) shellac-ethanol solution was added dropwise into 36 mL of each polyelectrolyte solution (SA, Gel, or CS at different concentrations) while stirring at 600 rpm and 25 °C. After 30 min of continuous stirring, ethanol was eliminated using a rotary evaporator (N-1100V-WB, Shanghai Ailang Instrument Co., Ltd., Shanghai, China) at 50 °C and 50 rpm. The volume was then restored to 48 mL with ultrapure water to yield the nanoparticle dispersion. All samples were stored at 4 °C until further analysis.

### 2.4. Experimental Methods

#### 2.4.1. Particle Size

The particle size and polydispersity index (PDI) of the samples were analyzed based on the procedure reported by Feng et al. [19] with minor adaptations. Measurements were conducted on a Nano-ZS90 Zetasizer (Malvern Instruments Co., Ltd., Malvern, Worcestershire, UK). Prior to analysis, the nanoparticle dispersion underwent a tenfold dilution with deionized water to minimize multiple light scattering effects. A 1 mL aliquot was transferred to a sample cell for measurement under the following conditions: sample refractive index of 1.52, dispersion medium water (refractive index 1.333), detection angle of 90°, equilibration time of 120 s, and 60 measurement runs.

#### 2.4.2. Zeta Potential

Zeta potential of the nanoparticles was measured following the approach outlined by Li et al. [18] with minor adjustments. A Nano-ZS90 Zetasizer (Malvern Instruments Co., Ltd., Malvern, Worcestershire, UK) was utilized to evaluate the zeta potential via dynamic light scattering. Specifically, 1 mL of the nanoparticle dispersion was transferred to a dedicated zeta potential cell. Measurements were performed after an equilibration period of 120 s at a constant temperature of 25 °C.

#### 2.4.3. Turbidity

The turbidity of the nanoparticle suspension was analyzed following the principle described by Cai et al. [20] with some adaptations. A 1 mL aliquot of the prepared nanoparticle solution was diluted tenfold with deionized water. Absorbance of the diluted sample was then measured at 600 nm using a SpectraMax M2 microplate reader (Molecular Devices, San Jose, CA, USA).

#### 2.4.4. pH Stability

The pH stability of the nanoparticle dispersions was evaluated according to a reported method with modifications Liang et al. [21]. The pH of each sample was adjusted to 6.0, 6.5, 7.0, and 7.5 using 0.1 M HCl or NaOH. Visual appearance was documented under standardized imaging conditions in a light box using a smartphone camera. The particle size, PDI, zeta potential, and turbidity (OD_600_) were measured after 10-fold dilution with ultrapure water.

### 2.5. Preparation of Cinnamon Essential Oil-Loaded Shellac Nanoparticles

CEO solutions at different concentrations (2%, 4%, 8%, *v*/*v*) were prepared using a 2% Tween-80 solution (*w*/*v*). Then, 12 mL of CEO solution was slowly added to the SA-SNPs/CS-SNPs dispersion under magnetic stirring at 600 rpm, 25 °C for 30 min. Ethanol was subsequently removed using a rotary evaporator at 50 °C and 50 rpm. The volume lost due to ethanol evaporation was replenished with ultrapure water to obtain a final nanoparticle dispersion of 60 mL. Subsequently, the obtained nanoparticle dispersion was centrifuged at 4 °C and 10,000 rpm for 30 min (3-30KS, Sigma, Darmstadt, Germany) to sediment the nanoparticles. The supernatant was discarded to remove unencapsulated CEO and free surfactants, and the nanoparticle pellet was re-dispersed in 60 mL of ultrapure water via vortex mixing and brief sonication. The purified nanoparticle dispersion was stored at 4 °C for further analysis. The resulting dispersions were stored at 4 °C for further analysis. Particle size, PDI, and zeta potential were determined as described in Section 2.4.1 and Section 2.4.2.

#### 2.5.1. Encapsulation Efficiency

The determination of encapsulation efficiency was carried out according to the method reported by Li et al. [18] with minor adjustments. The nanoparticle dispersion was centrifuged (3-30KS, Sigma, Darmstadt, Germany) at 10,000 rpm and 4 °C for 30 min to sediment the nanoparticles. Subsequently, 1 mL of the supernatant was collected, diluted ten-fold with purified water, and its absorbance was measured at 330 nm using a microplate reader. The concentration of free CEO was determined from the absorbance value using the standard curve. The encapsulation efficiency was calculated according to the following formula:

$$EE\;(\%)=\frac{W_{0}-W_{1}}{W_{0}}\times 100$$

where *EE*% is the encapsulation rate, *W*_0_ is the total essential oil mass (mg), and *W*_1_ is the mass of unencapsulated essential oil in the supernatant (mg).

#### 2.5.2. Fourier Transform Infrared (FT-IR) Spectroscopy

FT-IR spectra of the nanoparticle dispersions were conducted right after preparation on a Nicolet iN10 spectrometer (Thermo Fisher Scientific, Waltham, MA, USA) with an ATR accessory. Spectra were collected over the range of 4000–400 cm^−1^ at a resolution of 4 cm^−1^, averaging 32 scans per measurement.

#### 2.5.3. pH-Responsive Release

The pH-responsive release behavior of the nanoparticles was evaluated based on the procedure reported by Meng et al. [22], with slight adaptations. To simulate the pH environment associated with spoilage in aquatic products, phosphate-buffered saline (PBS) solutions with pH values of 6.2, 6.4, 6.6, 6.8, and 7.0 were prepared, each containing 0.5% (*w*/*v*) Tween-80 as the release medium. A 3 mL aliquot of the freshly prepared nanoparticle suspension was sealed in a dialysis bag and immersed in 50 mL of release medium. The system was then agitated at 150 rpm in a temperature-controlled shaker maintained at 30 °C. At designated time points (1, 2, 4, 6, 8, 10, 12, 24, 36, 48, 72, and 96 h), 1 mL of the release medium was sampled and simultaneously replenished with an equal volume of fresh buffer. The amount of released CEO was determined by measuring the absorbance at 330 nm using a microplate reader. The cumulative release percentage was calculated as follows:

$$M=\frac{C_{n}\times V_{0}+V_{i}\sum_{i=1}^{n-1}C_{i}}{m}\times 100\%$$

where *M* is the cumulative release rate, *C_n_* is the concentration of free CEO measured at a specific time point *n*, mg/mL. *V*_0_ is the total volume of dissolution medium, mL. *V_i_* is the volume of 1 mL of release medium withdrawn during a given time interval, mL. *C_i_* is the release concentration of CEO in the solution at time i, mg/mL. *m* is the initial encapsulation of total amount of CEO in the nanoparticles, mg.

The release kinetics of CEO from nanoparticles were analyzed using the zero-order model, first-order model, Higuchi model, and Ritger–Peppas model. The release models areas follows:

$$\mathrm{zero}\text{-}\mathrm{order}\;\mathrm{release}\;\mathrm{kinetic}\;\mathrm{model}:\;\frac{M_{t}}{M_{\infty }}=kt$$

$$\mathrm{first}\text{-}\mathrm{order}\;\mathrm{release}\;\mathrm{kinetic}\;\mathrm{model}:\;\frac{M_{t}}{M_{\infty }}=1-e^{-kt}$$

$$\mathrm{Higuchi}\;\mathrm{model}:\;\frac{M_{t}}{M_{\infty }}={kt}^{1/2}$$

$$\mathrm{Ritger}–\mathrm{Peppas}\;\mathrm{model}:\;\frac{M_{t}}{M_{\infty }}={kt}^{n}$$

where *M_t_* is the cumulative amount of CEO released at time *t*, *M*_∞_ is the cumulative amount of CEO released at equilibrium. *n* is the release exponent indicating the nature of the release mechanism. *k* is the kinetic constant (release rate constant).

### 2.6. Environmental Stability

#### 2.6.1. Storage Stability

The prepared nanoparticle dispersion was stored at 4 °C for 28 days. The average particle size, zeta potential, and PDI of the nanoparticle solution were measured at days 0, 7, 14, 21, and 28.

#### 2.6.2. Ionic Strength Stability

The ionic strength stability of the nanoparticles was evaluated by adding NaCl to freshly prepared dispersions to achieve final concentrations of 25, 50, 100, 200, and 300 mmol/L. After stirring thoroughly, the mixtures were equilibrated at room temperature for 3 h. The particle size, PDI, and zeta potential were then measured.

#### 2.6.3. Temperature Stability

The thermal stability of the nanoparticle dispersions was evaluated by storing them at −18 °C, 4 °C, and 25 °C for 24 h. Post-storage, a 2 h equilibration period at room temperature was implemented preceding the analysis of particle size and PDI.

### 2.7. Determination of Antibacterial Properties

The antibacterial activity was determined using a slightly modified version of the method described by Tan et al. [23]. The agar well diffusion assay was employed to evaluate the inhibitory activity of the nanoparticles against *Shewanella putrefaciens* and *Pseudomonas fluorescens*. Briefly, each bacterial strain was transferred to LB liquid medium and cultured at 30 °C with shaking (120 rpm) for 12 h to reach the logarithmic phase. A 100 μL volume of the bacterial suspension, adjusted to approximately 10^8^ CFU/mL, was then evenly spread on the solid medium surface. Wells with a diameter of 6 mm were created in the agar plates using a sterile puncher, and 100 µL of each sample solution was added into the wells. Following incubation at 30 °C for 24 h, the diameters of the inhibition zones were recorded.

### 2.8. Statistical Analysis

Each experiment was conducted with three independent replicates. Data are presented as mean ± standard deviation. Statistical comparisons between groups were evaluated through one-way analysis of variance (ANOVA) and Duncan’s multiple range test, with statistical significance defined as *p* < 0.05. All graphical representations were plotted using Origin 2024 software (OriginLab Corp., Northampton, MA, USA).

## 3. Results and Discussion

### 3.1. Effect of Different Polyelectrolytes on the Stability of Shellac Nanoparticles

#### 3.1.1. Particle Size

**Figure 2:**
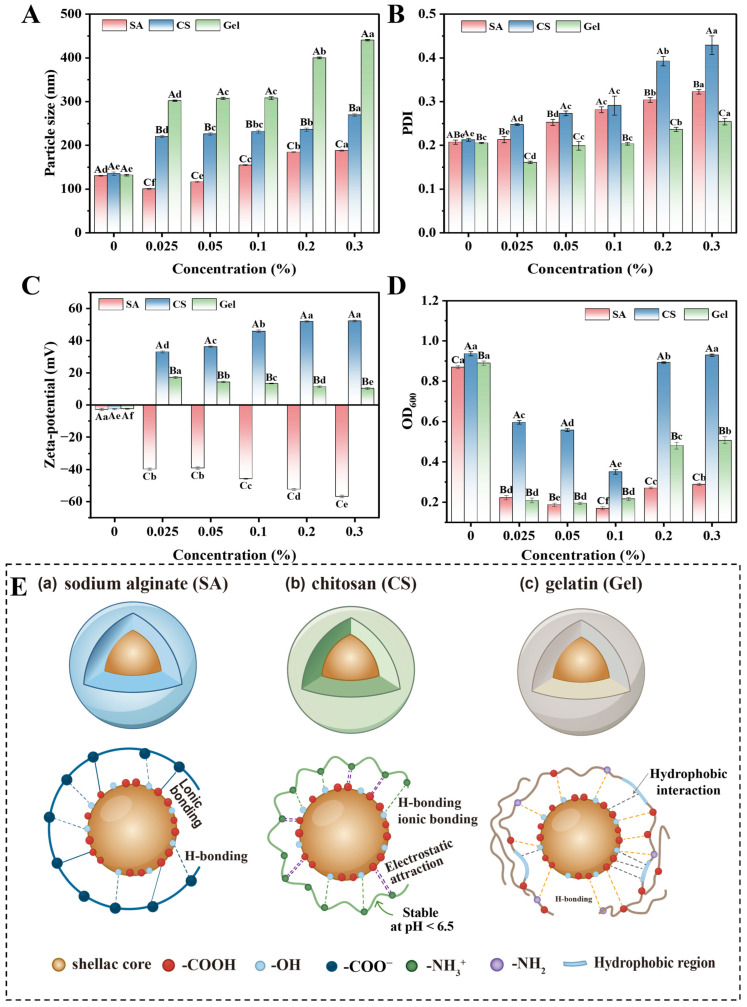
Particle size (nm) (**A**), PDI (**B**), zeta potential (mV) (**C**), turbidity (**D**) and mechanism diagram (**E**) of shellac nanoparticles (SNPs) modified by different types and modification concentrations of polyelectrolyte. SA: sodium alginate, CS: chitosan; Gel: gelatin. Different capital letters indicate statistically significant differences between samples of different types at the same modification concentration (*p* < 0.05), and different lowercase letters indicate statistically significant differences between samples of the same type at different modification concentrations (*p* < 0.05).

Particle size served as a critical indicator for assessing the physical stability of nanoparticles, directly influencing their dispersion and bioavailability. Shellac’s amphiphilic nature, stemming from its hydrophilic groups (-COOH, -OH) and hydrophobic regions, enables nanoparticle formation in aqueous systems but also predisposes it to hydrophobic aggregation via non-polar segment interactions [24,25]. As shown in Figure 2A,B, the nanoparticle particle size and PDI of all treatment groups showed an increasing trend with increasing polyelectrolyte concentrations. This can be attributed to three main interaction forces in the polyelectrolyte-SNP system (Figure 2E): the hydrophobic force between the shellac molecules attracting each other through the hydrophobic cores, the interfacial force where the specific functional groups of the polyelectrolyte form electrostatic, ionic, or hydrogen bonds with the corresponding groups on the surface of the shellac [26], and the flocculating force between the chains of the molecules of the excess polyelectrolyte, which is formed through entanglement and bridging [27]. When the polyelectrolyte addition is low, the hydrophobic force between the shellac molecules is dominant in the system. At this stage, the polyelectrolyte primarily forms an incomplete coating through physical adsorption and spatial occupation of molecular chains [28]. This interaction is relatively weak and struggles to effectively counteract the strong hydrophobic aggregation tendency within the shellac core. For CS and Gel, the particle size and PDI increased compared to those of the SNPs. In contrast, SA, owing to its linear rigid chain structure and high density of ionizable carboxyl groups, provides significant electrostatic repulsion even at low concentrations (*p* < 0.05) [29]. This effectively dispersed the nanoparticles, resulting in a marked reduction in their particle size. When the polyelectrolyte addition was appropriately increased, the interfacial force between the amphiphilic polyelectrolyte and shellac dominated the system. The regularly distributed carboxylate (-COO-) on the linear chain of SA and the polar groups on the surface of the shellac formed a composite force with strong electrostatic repulsion as the main force, supplemented by hydrogen bonding (Figure 2E(a)) [30], which effectively resisted the hydrophobic aggregation [31]. CS could effectively resist the hydrophobic aggregation through the protonated amino group (-NH_3_^+^) to form electrostatic attraction and ionic bonding with the negatively charged surface of shellac (Figure 2E(b)) [32]. Gel, on the other hand, physically adsorbs and encapsulates through multi-site hydrogen bonding and hydrophobic interaction (Figure 2E(c)). When polyelectrolyte is added in excess, bridging flocculation becomes the dominant mechanism, and its molecular chains cross-link the particles by entanglement, ultimately leading to a significant increase in particle size and PDI [27]. For the same polyelectrolyte addition, the SA showed the best performance, followed by CS, and Gel showed the relatively worst performance (*p* < 0.05). This can be explained by the linear, rigid chain structure of SA and its high density of carboxyl groups, which promote the formation of a stable interfacial layer via electrostatic repulsion, ultimately resulting in the smallest particle size and PDI [29]. Although CS can form a coating through ionic bonding, its PDI rises sharply with the concentration. The Gel mainly relies on physical adsorption, and its lower PDI reflects a homogeneous distribution of the large-sized aggregates rather than a good dispersion stability. Therefore, polyelectrolyte modification can effectively improve the stability of SNPs.

#### 3.1.2. Zeta Potential

Zeta potential is a key indicator of nanoparticle surface charge, with its absolute value directly related to colloidal stability. Dispersion systems with an absolute zeta potential > ±30 mV are generally considered stable [33]. As shown in Figure 2C, for a given polyelectrolyte, increasing its concentration caused the absolute zeta potential to first rise and then plateau. At lower concentrations, polyelectrolyte adsorption was insufficient to form a complete coating, leaving the shellac surface partially exposed. The ionization state of carboxyl and hydroxyl groups on shellac, influenced by solution pH, resulted in low surface charge density and poor system stability [15]. As the concentration increased to an optimal level, polyelectrolyte molecules formed a dense monolayer on the particle surface through electrostatic attraction, hydrogen bonding, and other interactions [34]. For SA, the high density of carboxyl groups (—COO^−^) became fully ionized under conditions far from its isoelectric point, generating strong electrostatic repulsion and significantly increasing the absolute zeta potential [35]. For CS, the protonated amino groups (—NH_3_^+^) reversed the zeta potential to positive via electrostatic attraction to the negatively charged shellac surface [36]. For Gel, as an amphoteric electrolyte with an isoelectric point (pI ≈ 4.8–5.2) near the experimental pH, its net charge density remained low, providing only weak electrostatic stabilization. At excessive concentrations, surface charge sites became saturated, stabilizing the zeta potential; for flexible polymers like CS, over-addition could also induce bridging flocculation, affecting measurements [37].

Comparing different polyelectrolytes at the same concentration revealed distinct effects. SA’s high density of carboxyl groups provided high negative charges, effectively increasing the absolute zeta potential (Figure 2E(a)) [35]. CS, rich in —NH_3_^+^ groups, reversed the zeta potential to positive via electrostatic attraction to the shellac surface, though its flexible chains and weak steric hindrance resulted in poorer double-layer stability (Figure 2E(b)). The gel, with its isoelectric point near the experimental pH, had a low net charge density, providing the weakest enhancement of zeta potential and relying mainly on hydrogen bonding and hydrophobic interactions for physical adsorption (Figure 2E(c)) [38].

Therefore, selecting the appropriate polyelectrolyte type and optimizing its concentration are essential for achieving optimal electrostatic stabilization.

#### 3.1.3. Turbidity

Turbidity (OD_600_) indicates the presence of large aggregates, with lower values signifying better dispersion and less aggregation [39]. The results showed that the type and concentration of polyelectrolyte had a decisive effect on the turbidity of the system (Figure 2D), and this result was consistent with the results of particle size and PDI.

For each polyelectrolyte, turbidity generally decreased to a minimum at an intermediate concentration before rising again at higher concentrations. At low concentrations, insufficient surface coverage failed to shield hydrophobic SNP cores, leading to high turbidity due to aggregation. At an optimal concentration, such as 0.1% for SA, a complete stabilizing layer formed, minimizing particle aggregation and achieving the lowest turbidity. The strong electrostatic repulsion from SA’s ionized carboxyl groups, combined with steric hindrance, effectively prevented particle coalescence [31,40]. Beyond this point, further concentration increase caused turbidity to rise due to bridging flocculation, where excess free polymer chains adsorbed onto multiple nanoparticles, forming large, light-scattering aggregates [41,42]. This increase was most pronounced for CS and Gel: for CS, the flexible chains increased the risk of charge neutralization and bridging at higher concentrations [36,43]; for Gel, the weak electrostatic stabilization and reliance on hydrogen bonding made it more susceptible to aggregation [44]. SA showed a more moderate rise, highlighting its superior stability even under non-optimal concentrations. SA-modified SNPs consistently showed the lowest turbidity, especially at 0.1%, due to its strong electrostatic repulsion and steric hindrance effects. CS-modified systems exhibited higher turbidity, as its flexible chains increased the risk of bridging flocculation at higher concentrations. Gel-modified systems showed the highest turbidity, as its stabilization relied predominantly on weak steric hindrance and hydrophobic interactions, which were insufficient to prevent aggregation effectively.

In conclusion, the turbidity analysis demonstrates that SA at 0.1% concentration achieves optimal clarity by combining strong electrostatic and steric stabilization, while excessive concentrations or suboptimal polyelectrolyte selection (CS or Gel) promote aggregation through bridging flocculation or weak charge effects.

#### 3.1.4. pH Stability

**Figure 3:**
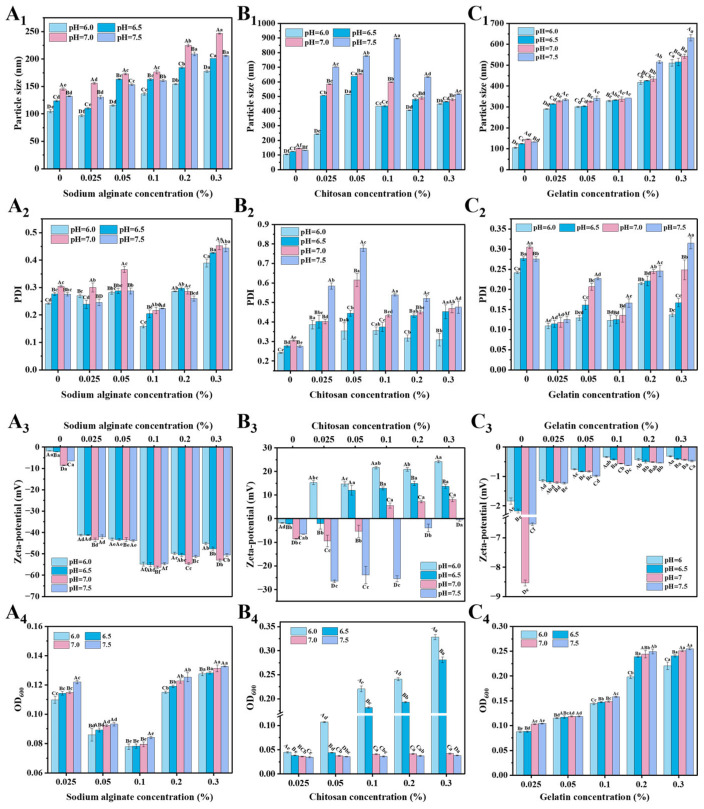
Effect of pH on particle size (nm), PDI, zeta potential (mV) and turbidity of nanoparticles. SA (**A_1_**–**A_4_**), CS (**B_1_**–**B_4_**), Gel (**C_1_**–**C_4_**). Different uppercase letters indicate statistically significant differences between samples with the same concentration and different pH treatments (*p* < 0.05), and different lowercase letters indicate statistically significant differences between samples with different concentrations under the same pH treatment (*p* < 0.05).

Nanoparticles are required to withstand pH changes during spoilage of aquatic products, thus their pH stability is critical. In this study, the stability of three modified nanoparticles was systematically evaluated in the pH range of 6.0–7.5, which mimics the typical pH changes during storage of aquatic products. The results showed that SNPs were poorly stabilized, whereas polyelectrolyte modification significantly improved their stability, but the improvement strongly depended on the type of polyelectrolyte (Figure 3 and Figure S1).

SA-SNPs showed excellent and consistent stability under all pH conditions with minimal changes in particle size, PDI and zeta potential. This was attributed to the low pKa values (~3.5–4.0) of the carboxyl group (-COOH) on the SA molecular chain. In the tested pH range (6.0–7.5), these carboxyl groups were almost completely ionized to negatively charged carboxylic acid radicals (-COO^−^), which provided a sustained and strong electrostatic repulsion on the particle surface [22], effectively resisting the aggregation tendency due to pH fluctuations. As a result, the turbidity of SA-SNPs was also consistently the lowest and most stable throughout the pH range (Figure 3A_1_–A_4_), which macroscopically showed a homogeneous and precipitation-free dispersion system (Figure S1A).

In contrast, CS-SNPs, whose stability exhibits a strong pH dependence. Under acidic conditions (pH 6.0, 6.5), the amino group on the CS chain is in a protonated state (-NH_3_^+^) and binds to the negatively charged shellac nucleus by strong electrostatic attraction to form a relatively stable complex with a positive zeta potential [45]. However, when the pH was raised close to or above the pKa of CS (≈6.5), the amino group underwent deprotonation (-NH_2_), resulting in the reduction or even disappearance of its positive charge. This not only weakens the electrostatic attraction between the CS and the shellac nucleus, but more critically, drastically reduces the electrostatic repulsion between particles, which triggers severe aggregation and precipitation [43,45]. This is directly manifested macroscopically by the sharp increase in solution turbidity at pH 7.0 and 7.5 (Figure 3B_1_–B_4_) and flocculation visible to the naked eye (Figure S1B). For Gel-SNPs, the stability is between SA and CS. Since the gelatin molecule contains both amino and carboxyl groups, its net charge does not vary much over the tested pH range, and it mainly relies on hydrogen bonding and hydrophobic interactions to provide some spatial stability (pKa ≈ 5.0). As a result, its particle size growth and PDI changes are more gentle relative to CS, but due to the lack of a strong electrostatic stabilization mechanism [44], its stabilization is much less effective than that of SA, and the turbidity values are consistently higher (Figure 3C_1_–C_4_ and Figure S1C). Karim et al. [45] and Chen et al. [46] also studied CS-based composite nanoparticle systems. It was demonstrated that variations in environmental pH enhanced the positive charge density of the materials via protonation, which altered the swelling behavior of the nanoparticles. This led to a notable growth in particle size, thereby promoting the release of the encapsulated material.

Based on the comprehensive screening of polyelectrolytes, SA and CS at a concentration of 0.1% (*w*/*v*) were identified as the two most promising modifiers for SNPs and were therefore selected for subsequent loading experiments with CEO. Although SA demonstrated superior overall stability, CS was chosen alongside it because it forms nanoparticles with a high positive zeta potential, representing a distinct and valuable characteristic for comparison. The difference in their charge profiles (anionic for SA, cationic for CS) leads to variations in key properties when interacting with CEO, such as essential oil loading capacity, encapsulation efficiency, and release behavior. Therefore, selecting both systems enables a comparative evaluation of how polyelectrolyte charge characteristics influence the performance of the resulting delivery systems.

### 3.2. Characterization of Cinnamon Essential Oil-Loaded Shellac Nanoparticles

#### 3.2.1. Particle Size

**Figure 4:**
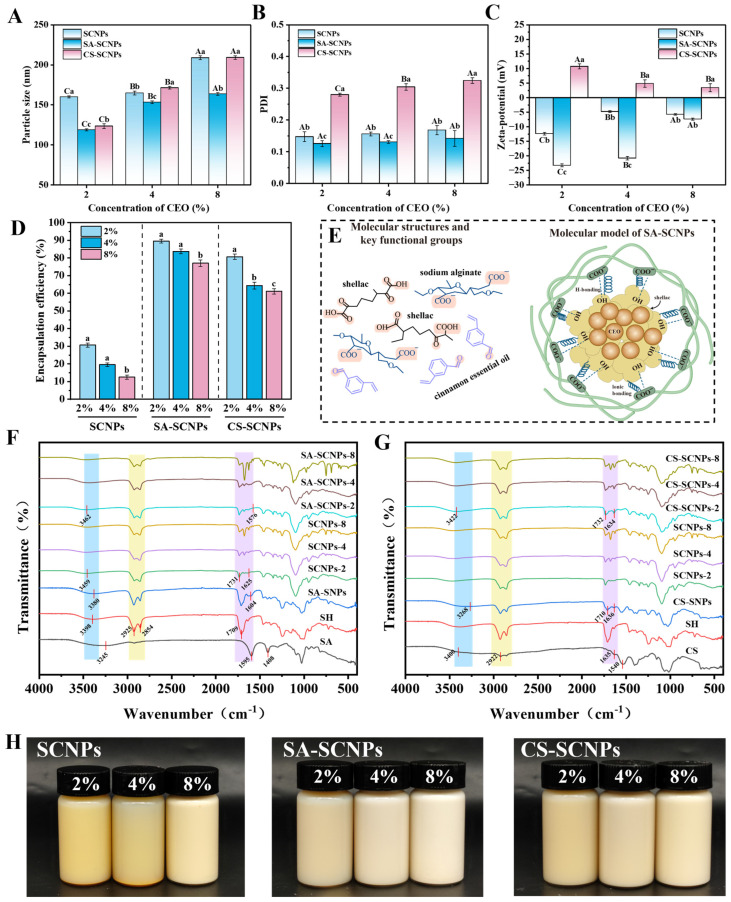
Particle size (nm) (**A**), PDI (**B**), zeta potential (mV) (**C**), encapsulation efficiency (%) (**D**), schematic diagram (**E**), FT-IR: SA-SCNPs (**F**), CS-SCNPs (**G**), and sample morphology (**H**). Different letters indicate significant differences (*p* < 0.05).

Particle size and PDI are critical parameters governing the stability and functionality of nanoparticles loaded with essential oils [47]. As shown in Figure 4A,B,H, both SA- and CS-modified nanoparticles showed significantly better size and PDI than unmodified SCNPs (*p* < 0.05), with SA-SCNPs performing best, having the smallest size and lowest PDI. Increasing CEO concentration from 2% to 8% caused particle size to grow in all systems, but to different degrees. This concentration-dependent expansion is due to increased oil volume and interfacial tension [48]. SA-SCNPs showed the most limited size increase, demonstrating high adaptability to oil loading. This is attributed to the strong electrostatic repulsion and steric hindrance provided by the SA layer, which effectively counteract the increased interfacial tension associated with higher oil loading [49,50]. In contrast, CS-SCNPs showed the greatest size expansion at high CEO concentrations. The flexible chains of CS are more prone to conformational rearrangement at the oil-water interface, leading to a less stable interfacial layer that is more easily disrupted under high oil loading [51,52,53].

In summary, both polyelectrolyte type and CEO concentration affect nanoparticle structure. SA-modified nanoparticles maintain optimal size across a wide CEO range (2–8%), showing superior adaptability. CS-based systems, however, have a limited capacity to maintain stability at higher loads. These findings highlight the importance of selecting the right polyelectrolyte and optimizing oil concentration for developing stable nanoencapsulation systems in food preservation.

#### 3.2.2. Zeta Potential

Zeta potential can reflect the repulsive force or attractive force between the surface charge of emulsion particle and more particles on the emulsion surface [54]. As shown in Figure 4C, SA-SCNPs exhibited the highest absolute zeta potential values across all CEO concentrations, significantly exceeding those of unmodified SCNPs (*p* < 0.05). This enhanced surface charge originates from the strongly ionized carboxylate groups densely distributed along the SA molecular chains, which provide sustained electrostatic repulsion and superior stabilization [35]. In contrast, CS-SCNPs displayed positive zeta potential values, consistent with the cationic nature of chitosan arising from its protonated amino groups [32]. As the CEO loading increased from 2% to 8%, a consistent decline in the absolute zeta potential was observed across all nanoparticle systems. This behavior can be attributed to two main factors: first, the adsorption of CEO components, particularly phenolic compounds, at the oil–water interface may partly obscure the surface charge of the nanoparticles [55]. second, the stabilization mechanism progressively shifts from electrostatic repulsion toward steric stabilization as the essential oil loading rises, thereby reducing the reliance on surface charge for colloidal stability [31,56]. This result was consistent with the findings of López-Meneses et al. [57], who reported a decrease in the zeta potential of nanoparticles after the encapsulation of *Schinus molle* essential oil. Notably, SA-SCNPs demonstrated the smallest reduction in zeta potential magnitude with increasing CEO concentration, maintaining a high absolute value even at the highest loading of 8%. This is due to the robust charge characteristics of SA, which effectively compensate for the charge shielding effect caused by CEO encapsulation [35]. In contrast, CS-SCNPs experienced a more pronounced decrease in zeta potential, indicating relatively weaker stability under high CEO concentrations. This more rapid decline can be attributed to several interrelated factors inherent to the CS system: first, the flexible chains of CS undergo conformational rearrangement at the interface as CEO concentration increases, reducing the effective exposure of protonated amino groups [58]; second, phenolic compounds in CEO interact with the amino groups of CS through hydrogen bonding and hydrophobic interactions, leading to partial charge neutralization [59]; third, the system pH (≈6.5) is close to the pKa of chitosan, making the charge status of CS particularly susceptible to minor environmental changes induced by CEO incorporation. The combination of these factors makes the CS-based system more vulnerable to charge shielding and structural reorganization under high oil loading.

In summary, the SA modification shows the highest absolute value of zeta potential and maintains excellent stability even at high essential oil concentrations.

#### 3.2.3. Encapsulation Efficiency

Encapsulation efficiency is a key parameter used to evaluate the effective encapsulation of various bioactive substances by nanoparticles [60]. As shown in Figure 4D, compared with SCNPs, modification with SA or CS significantly increased the encapsulation efficiency. This may be attributed to the fact that the hydrophobic shellac surface alone is insufficient to form a stable and elastic barrier at the oil–water interface, potentially leading to leakage of encapsulated CEO [61]. The significant enhancement in encapsulation efficiency with SA or CS modification is mainly due to the formation of a polyelectrolyte interfacial layer. As hydrophilic polymers, SA and CS adsorb at the interface between the hydrophobic core and the aqueous medium by reducing interfacial energy and leveraging the amphiphilic nature of their molecular chains [62]. The adsorbed polymers form a thicker, viscoelastic film that provides enhanced steric hindrance, effectively preventing the coalescence and leakage of essential oil droplets [63,64]. Secondly, as the concentration of CEO increases, the encapsulation efficiency of all three groups of nanoparticles shows a significant decreasing trend. This indicates that the encapsulation capacity of nanoparticles has a saturation limit, excess free CEO adheres to the nanoparticle surfaces, reducing electrostatic repulsion and thereby inducing nanoparticle aggregation and precipitation [65,66]. The results of this study are consistent with the research by [67], where the encapsulation efficiency of chitosan nanoparticles gradually decreased as the content of ginger essential oil increased. Furthermore, the encapsulation efficiency of SA-SCNPs was consistently higher than that of CS-SCNPs. This performance difference can be understood from the distinct physicochemical properties of the two polymers. SA possesses a high density of ionized carboxyl groups under the experimental conditions, which promotes the formation of a dense, cohesive interfacial layer around the oil droplets through strong electrostatic repulsion and chain rigidity. This robust interfacial layer effectively encapsulates CEO and prevents its leakage [68,69]. In contrast, the more flexible chains of CS tend to form a less densely packed interfacial layer with lower mechanical strength, making it less effective at retaining CEO under high loading conditions, although it still provides significant improvement over unmodified SCNPs.

In summary, while both SA and CS modifications improve encapsulation efficiency, SA-based nanoparticles demonstrate optimal performance at a CEO concentration of 2–4%, beyond which a notable decline in encapsulation efficiency is observed. These findings suggest that 2–4% CEO represents the optimal loading range for achieving high encapsulation efficiency while maintaining nanoparticle stability.

#### 3.2.4. FT-IR

The interactions among various components in nanoparticles were examined using FT-IR spectroscopy (Figure 4F,G), Analysis of the infrared spectra revealed that shellac (SH) corresponds to the O-H stretching vibration at 3398 cm^−1^, with C-H stretching vibration peaks at 2925 cm^−1^ and 2854 cm^−1^, as well as an ester carbonyl (C=O) peak at 1709 cm^−1^ [70]. SA displayed a broad hydroxyl peak at 3245 cm^−1^, along with asymmetric and symmetric stretching vibration peaks for the carboxylic acid salt (-COO^−^) at 1595 cm^−1^ and 1408 cm^−1^ [71]. The broad absorption bands near 3400 cm^−1^ in the CS spectrum arise from O–H and N–H stretching vibrations, while the peak at 2922 cm^−1^ is attributed to C–H stretching of methyl/methylene groups. Furthermore, a C=O stretching vibration corresponding to the amide I band appears around 1635 cm^−1^, and the bending vibration of the amino group is observed at 1540 cm^−1^ [72].

In the FT-IR spectra of SA-SNPs, the carbonyl peak of shellac at 1709 cm^−1^ completely disappeared, while the asymmetric stretching vibration peak of the carboxylate of SA was significantly displaced from 1594 cm^−1^ to 1604 cm^−1^. The displacement of this characteristic peak indicates the formation of ionic and hydrogen bonds between the -COO^−^ of SA and the -COOH group of shellac [71] (Figure 4E). The carbonyl peak of shellac in CS-SNPs was significantly attenuated and enhanced by merging with the amide I band (1635 cm^−1^) of CS, and the O-H/N-H stretching vibration peak was red-shifted to 3403 cm^−1^, indicating that surface modification between the amino group of CS and shellac was achieved by electrostatic attraction and hydrogen bonding network [73]. Upon CEO loading into the polysaccharide-coated nanoparticles, further evidence of hydrogen bonding was observed in the 1000–1200 cm^−1^ region, which reflects structural vibrations of monosaccharide residues and C–OH bonds. Specifically, at 1030 cm^−1^ and 1080 cm^−1^, corresponding to C–O stretching and C–OH bending vibrations of polysaccharides, the peak intensities were significantly reduced in SA-SCNPs and CS-SCNPs compared to unmodified SCNPs. Additionally, the band near 1150 cm^−1^, attributed to asymmetric stretching of C–O–C in glycosidic linkages, exhibited broadening and a slight redshift. These spectral changes indicate the formation of hydrogen bonds between the hydroxyl groups of the polysaccharide coatings and the functional groups of shellac or the encapsulated CEO. These results provide direct evidence that both SA and CS were successfully adsorbed onto the shellac surface via intermolecular interactions, with the nature of the interaction varying according to the functional groups present.

The effectiveness of CEO encapsulation was further evaluated by examining the characteristic CEO peak at 1675 cm^−1^. The ATR-IR spectra were normalized to enable a rigorous comparison of peak intensities. In unmodified SCNPs, the intensity of this peak increased significantly with rising CEO concentration, indicating inadequate encapsulation and surface adsorption or leakage of CEO molecules. In contrast, both SA-SCNPs and CS-SCNPs exhibited a substantial reduction or complete disappearance of the CEO characteristic peak, with minimal changes in intensity as the CEO concentration increased. This demonstrates that the polyelectrolyte modification formed a compact protective shell around the shellac core, effectively encapsulating the CEO within the nanoparticle interior and shielding its vibrational signals (Figure 4E).

In conclusion, FT-IR analysis verified that modification with SA and CS promoted the formation of a core–shell architecture via ionic, hydrogen, and electrostatic interactions. SA demonstrated superior encapsulation efficiency and structural integrity, which can be attributed to its higher density of functional groups and stronger interfacial interactions.

#### 3.2.5. pH-Responsive Release

**Figure 5:**
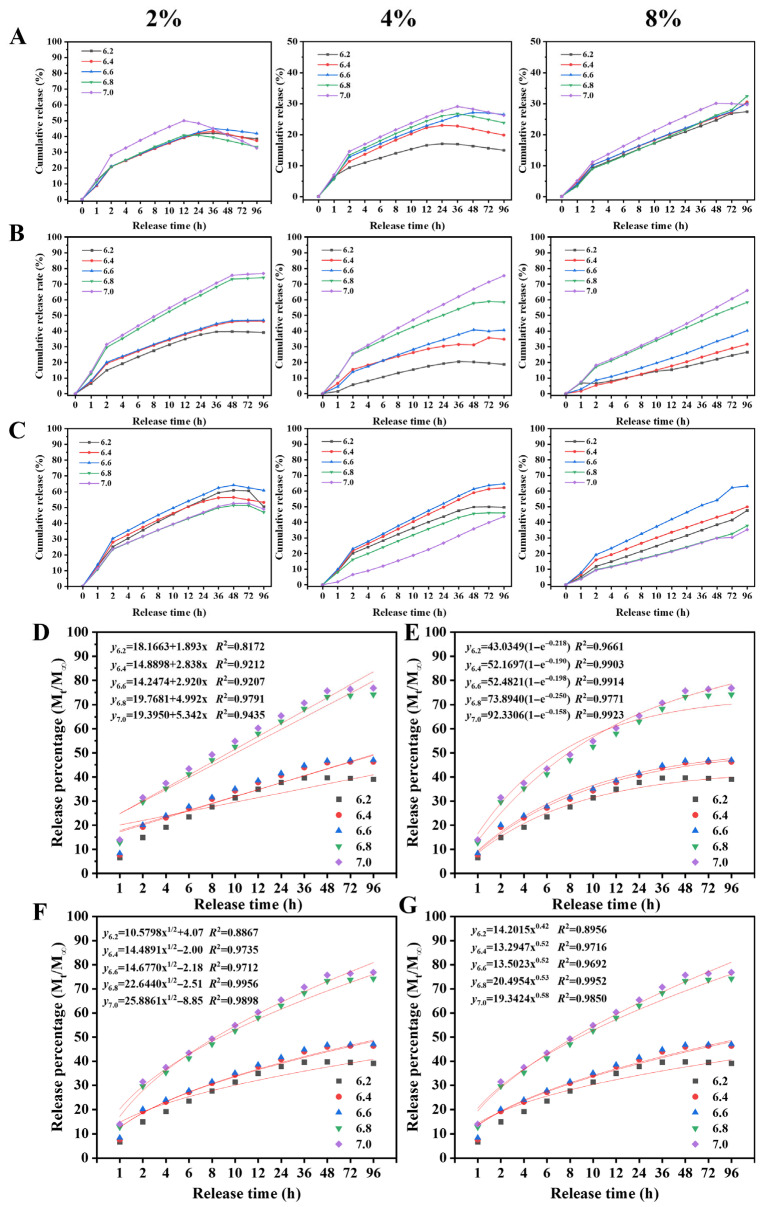
Cumulative release rate of CEO from SA-SCNPs under different pH conditions and fitting of release kinetic models. Cumulative release rates under different pH conditions (**A**–**C**): SCNPs (**A**), SA-SCNPs (**B**), CS-SCNPs (**C**). The figures represent loading concentrations of 2%, 4%, and 8% for CEO. Release kinetic models for SA-SCNPs-2 (**D**–**G**). Zero-order release model (**D**), First-order release model (**E**), Higuchi release model (**F**), and Ritger–Peppas release model (**G**).

The release profiles of CEO from shellac-based nanoparticles were systematically investigated under pH conditions simulating the spoilage process of aquatic products (pH 6.2–7.0). As shown in Figure 5A–C, both SA and CS modifications significantly enhanced the pH-responsive release properties compared to unmodified SCNPs across the entire pH range. While all formulations showed increased release rates with rising pH, SA-SCNPs exhibited the most pronounced response, particularly under alkaline conditions (pH ≥ 6.8). The release kinetics displayed characteristic biphasic patterns consisting of an initial rapid release phase (2–12 h) followed by a sustained release plateau. This pattern was observed across all pH conditions but became more pronounced as pH increased. The initial burst release is attributed to the rapid diffusion of surface-adsorbed or loosely bound CEO molecules [74], while the sustained phase is governed by the slower diffusion from the nanoparticle core [75]. The duration and intensity of both phases showed clear pH dependence, with higher pH values accelerating both the initial release rate and the subsequent sustained release.

Comparativeanalysis revealed fundamental differences in the release mechanisms between SA- and CS-modified nanoparticles. For SA-SCNPs, the ionization of carboxyl groups (–COOH → –COO^−^) with increasing pH generated strong electrostatic repulsion, leading to matrix swelling and pore formation that facilitated CEO diffusion [76]; at a higher pH, this swelling eventually contributed to gradual matrix erosion, further promoting release. This mechanism resulted in progressively faster release rates across the entire pH range (6.2–7.0). In contrast, CS-SCNPs exhibited optimal release performance in the pH 6.2–6.6 range, where the amino groups remain protonated. However, as the pH approached and exceeded 6.5 (near the pKa of chitosan), deprotonation caused structural instability and particle aggregation, creating a more tortuous diffusion path that compromised release efficiency at higher pH values [45,77].

To elucidate the release mechanisms, the release data were fitted to four kinetic models (Figure 5D–G). Given the superior pH responsiveness of SA-SCNPs and their relevance to spoilage conditions, the following kinetic analysis focuses on this formulation. The Zero-order model showed poor fitting at pH 6.2 (*R*^2^ = 0.817) but improved significantly at higher pH values (*R*^2^ = 0.979 at pH 6.8), indicating a transition toward constant-rate release near spoilage conditions. The consistent excellent fit of the First-order model (*R*^2^ > 0.966 across all pH levels) confirmed the diffusion-dominated nature of the release process. The Higuchi model provided good correlation at elevated pH, supporting the diffusion-based release mechanism. Most importantly, the Ritger–Peppas model revealed a crucial mechanistic insight: the release exponent (*n*) increased from 0.42 at pH 6.2 to 0.58 at pH 7.0, indicating a transition from Fickian diffusion toward a combination of diffusion and polymer matrix erosion mechanisms. This *n*-value progression aligns with the proposed mechanism that matrix swelling at higher pH gradually evolves into erosion, thereby providing quantitative evidence that the release mechanism shifts from pure diffusion to a more complex erosion-controlled process as pH increases. The kinetic parameters further confirmed the pH-triggered release behavior, with the first-order rate constant increasing from 43.03 to 92.33 as pH rose from 6.2 to 7.0. This systematic enhancement of release kinetics with increasing pH demonstrates the excellent pH-responsive characteristics of the developed nanoparticle system.

This intelligent, spoilage-responsive release profile represents a distinct strategy compared to high-performance sustained-release systems based on diffusion barriers. For instance, composite nanoparticle systems, such as soy protein isolate/tea saponin nanoemulsions, can provide excellent long-term stability and controlled release primarily through passive diffusion [78]. In such systems, release kinetics are largely time-dependent and lack active responsiveness to environmental cues. In contrast, the SA-SCNPs system leverages the inherent pH-sensitivity of shellac to introduce an active trigger mechanism. This design shifts the release paradigm from a passive, time-dependent process to an active, condition-responsive release that is precisely aligned with the spoilage dynamics of aquatic products, offering a more targeted delivery strategy.

The intelligent release profile of SA-SCNPs aligns closely with the dynamic spoilage process of aquatic products, particularly fish. During storage, the pH of fresh fish typically rises from around neutral (∼6.2–6.5) to alkaline (>7.0) due to the production of basic compounds (e.g., ammonia, trimethylamine) by spoilage bacteria such as *Shewanella putrefaciens*. The SA-SCNPs system, with its significantly higher cumulative release at pH 7.0 (77.76%), is designed for “on-demand” release. This means minimal CEO is released during the initial storage phase when microbial activity is low, preserving the active compound. Notably, the first-order release rate constant at pH 7.0 (92.33) substantially exceeds that at pH 6.2 (43.03), suggesting that CEO release accelerates precisely during the exponential growth phase of spoilage microorganisms. Once spoilage commences and the pH increases, a burst of CEO is triggered to target the proliferating microbial population. This responsive mechanism addresses the limitation of conventional preservatives that often exhibit premature burst release, offering a more precise and efficient strategy for extending shelf-life.

In summary, the release study demonstrates that SA-modified nanoparticles exhibit superior pH-responsive release characteristics across the entire pH range relevant to aquatic product spoilage. The combination of pH-dependent release profiles and well-fitted kinetic models confirms that SA-SCNPs undergo a mechanism transition from diffusion-dominated to erosion-assisted release as pH increases, making them ideal carriers for intelligent food preservation applications where controlled release in response to spoilage-induced pH changes is required.

#### 3.2.6. Environmental Stability

##### Storage Stability

**Figure 6:**
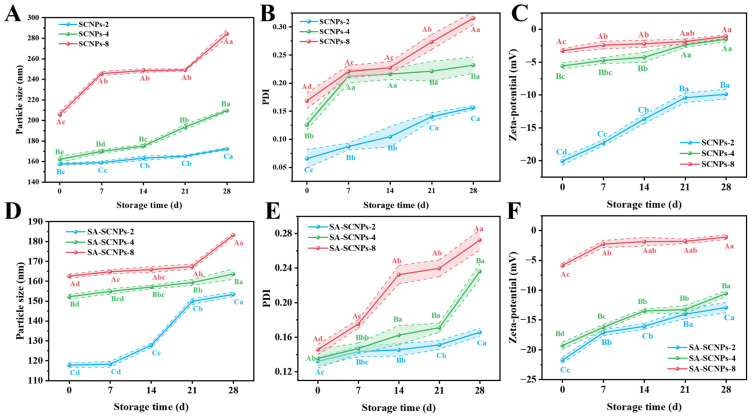
Storage stability of SCNPs (**A**–**C**), SA-SCNPs (**D**–**F**). Different capital letters indicate statistically significant differences (*p* < 0.05) between samples with different concentrations of the same storage time, and different lower case letters indicate statistically significant differences (*p* < 0.05) between samples with different storage times of the same sample.

Excellent storage stability is a prerequisite for the practical application of nanocarriers. As shown in Figure 6, SA-SCNPs exhibited far better stability than unmodified SCNPs at all CEO loading concentrations throughout the storage period, with a significantly smaller increase in particle size versus PDI (*p* < 0.05). This enhanced stability stems from the synergistic effect of kinetic and thermodynamic stabilization provided by SA modification. First, the hydrophilic layer formed by SA molecules on the particle surface constitutes a physical barrier with significant spatial site resistance effect through strong hydration [79], which effectively slows down the collision and aggregation of particles due to brownian motion [31]. Secondly, the high negative zeta potential (Figure 4C) conferred to the particles by the SA chains provides a strong electrostatic repulsion [80], which thermodynamically improves the energy barrier for the aggregation of the system.

With the increase in CEO concentration, the particle size of SA-SCNPs increased slowly from 153.33 nm to 183.13 nm, with a much smaller change than that of SCNPs. This confirms that the mechanical strength of the SA interfacial layer better buffers the interfacial tension due to the highly loaded cores [50]. The SA-SCNPs also showed a more gentle trend of zeta potential decrease, indicating a more durable surface charge layer [81]. Thus, SA-SCNPs exhibited superior storage stability compared to SCNPs. The excellent storage stability of SA-SCNPs at 4 °C for 28 days, with minimal changes in particle size and PDI, covers the typical shelf-life and distribution timeline for many refrigerated aquatic products. This robustness suggests that the nanoparticles can be incorporated into preservation formats (e.g., as a dip or in a coating) before packaging and maintain their structural integrity and functional activity throughout the cold chain—from processing and transportation to retail display. This meets a fundamental requirement for commercial application, ensuring that the preservative system remains effective until the product reaches the consumer.

##### Ionic Strength Stability

Evaluating ionic strength stability is crucial for food preservation, especially for aquatic products which contain salts and undergo varying salt conditions during processing. High ionic strength can shield electrostatic charges and disrupt colloidal stability, making it essential to test nanoparticle robustness under such conditions.

**Figure 7:**
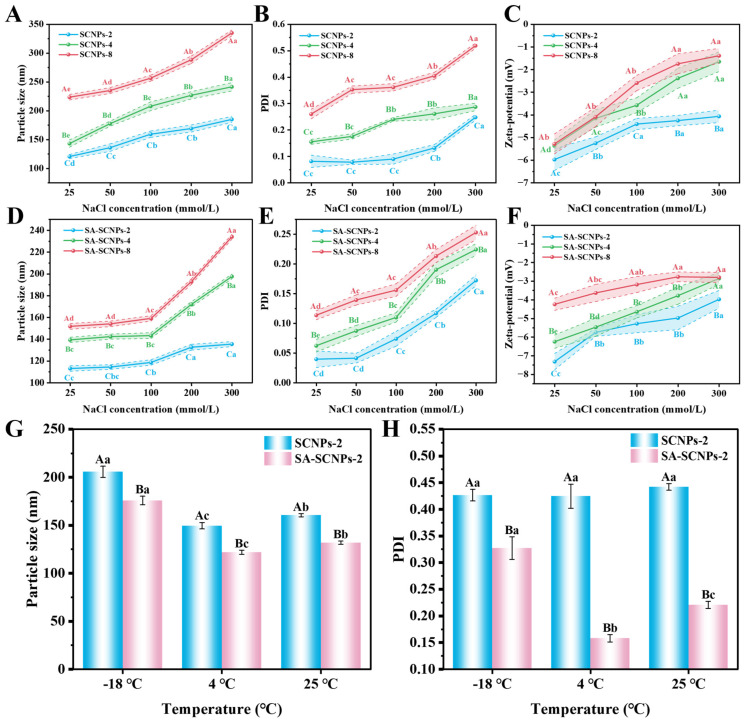
Stability characterization of SCNPs and SA-SCNPs. Changes in particle size (nm), PDI, and zeta potential (mV) of SCNPs (**A**–**C**) and SA-SCNPs (**D**–**F**) under different NaCl concentrations. Changes in particle size (**G**) (nm) and PDI (**H**) of SCNPs-2 and SA-SCNPs-2 at different storage temperatures. Different capital letters indicate significant differences (*p* < 0.05) among different samples under a fixed condition (NaCl concentration or temperature); different lowercase letters indicate significant differences (*p* < 0.05) within the same sample among different conditions (NaCl concentration or temperature).

As shown in Figure 7, SA-SCNPs (D–F) exhibited significantly better stability than unmodified Figure 7, SCNPs (A–C) across all tested ionic strengths (25–300 mmol/L NaCl) and CEO concentrations, demonstrated by smaller particle size and lower PDI (*p* < 0.05). This enhancement is due to SA’s dual stabilization mechanism. The dense SA layer on the shellac surface forms a compact structure that reduces the direct impact of NaCl [19,82], while its long chains provide steric hindrance against salt-induced aggregation. With increasing ionic strength, all systems showed increased particle size and PDI, but SA-SCNPs exhibited a much more moderate growth trend. This is due to the electrostatic shielding effect, where counter-ions (Na^+^, Cl^−^) neutralize nanoparticle surface charge [83,84], compressing the electrical double layer and reducing inter-particle repulsion. SA’s steric hindrance significantly mitigates this effect, maintaining stability even at high salt concentrations.

Zeta potential results provided further insight. SA-SCNPs maintained significantly higher absolute zeta potential values than SCNPs across all conditions (*p* < 0.05), indicating stronger electrostatic stabilization. Although zeta potential decreased with increasing ionic strength due to charge shielding, the decrease was significantly smaller for SA-SCNPs. This demonstrates that SA’s spatial stabilization plays a crucial synergistic role when electrostatic forces are weakened.

The superior performance of SA-SCNPs under high ionic strength is attributed to sodium alginate’s unique properties. Its linear rigid chains form a dense protective layer providing both electrostatic and steric stabilization. The high carboxyl group density maintains strong surface charge, while the polymer chains create physical barriers against aggregation [85]. This dual mechanism ensures structural integrity in high-ionic-strength environments relevant to aquatic products.

In summary, SA modification significantly enhances the ionic strength stability of SNPs through synergistic electrostatic and steric stabilization, demonstrating excellent potential for applications in salt-containing food systems. The stability of SA-SCNPs under high ionic strength (up to 300 mmol/L NaCl) is highly relevant for aquatic product applications. Seafood possesses inherent ionic content, and processing steps may involve brining, saline washing, or contact with marinades. The resistance of SA-SCNPs to aggregation under such conditions, primarily due to the steric hindrance provided by the SA layer, indicates that the delivery system can withstand the intrinsic ionic environment of the food matrix and mild processing conditions. This ensures that the nanoparticles’ functionality (e.g., antibacterial activity) is not compromised in real, salt-containing seafood products, facilitating their practical deployment.

##### Temperature Stability

Temperature stability is a critical requirement for nanocarriers used in food applications, as they may encounter varying storage conditions ranging from frozen to ambient temperatures. As illustrated in Figure 7G,H, SA-SCNPs-2 exhibited excellent stability at both refrigeration (4 °C) and room temperature (25 °C) conditions, with significantly smaller particle size and lower PDI values compared to unmodified SCNPs-2 (*p* < 0.05). At 4 °C, the PDI of SA-SCNPs reached an optimal value, indicating a near-monodisperse distribution. This can be attributed to the reduced Brownian motion at lower temperatures combined with the enhanced steric stabilization provided by the SA layer, which collectively minimize particle aggregation [86]. Under freezing conditions (−18 °C), all nanoparticle systems showed an increase in particle size due to the mechanical stress induced by ice crystal formation during the freezing process [73]. However, SA-SCNPs demonstrated significantly better cryostability, with a much smaller size increase compared to SCNPs. This superior performance can be explained by the cryoprotective effect of the SA layer. The hydrophilic nature of SA enables it to form strong hydrogen bonds with water molecules, thereby inhibiting ice crystal nucleation and modifying crystal growth patterns [87]. This interaction reduces the mechanical damage to the nanoparticle structure during freeze–thaw cycles, maintaining system integrity. Collectively, these temperature-dependent behaviors reveal that SA modification provides effective stabilization through multiple mechanisms: at refrigerated conditions, it maintains colloidal stability by combining reduced particle kinetics with steric hindrance; at freezing temperatures, it offers cryoprotection through ice crystal modulation; and at room temperature, it ensures stability via strong electrostatic and steric repulsion.

In summary, SA-modified nanoparticles exhibit excellent temperature adaptability, maintaining structural stability across a wide temperature range relevant to food storage conditions, which underscores their potential for practical applications in aquatic product preservation. The broad-temperature stability of SA-SCNPs (−18 °C to 25 °C) is crucial for practical preservation scenarios that encompass the entire supply chain. Frozen storage (−18 °C) is common for long-term preservation of raw fish, while chilled storage (4 °C) is used for fresh or ready-to-eat products. The limited particle growth observed at −18 °C, attributed to the cryoprotective effect of SA, suggests the system can survive freeze–thaw cycles, making it suitable for frozen products. Furthermore, stability at 4 °C and 25 °C ensures effectiveness during refrigeration and potential temperature fluctuations during distribution or retail. This wide adaptability enhances the system’s reliability and practicality for diverse preservation applications.

### 3.3. Antibacterial Activity Analysis

**Figure 8:**
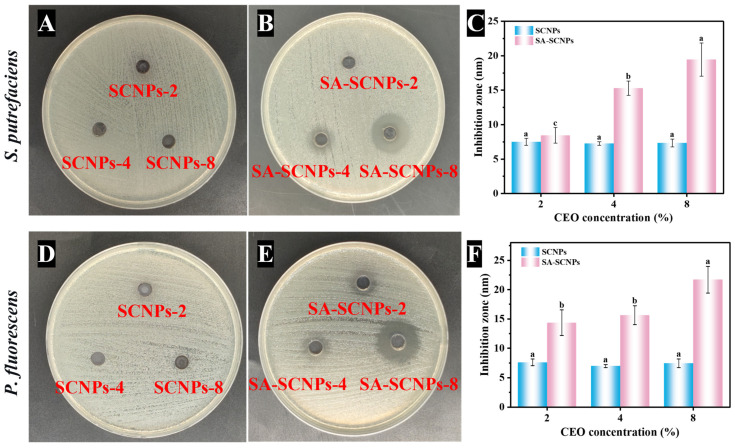
Antibacterial efficacy of CEO-loaded nanoparticles against *Shewanella putrefaciens* (**A**–**C**) and *Pseudomonas fluorescens* (**D**–**F**). Different letters indicate significant differences (*p* < 0.05).

Antibacterial properties are important indicators for evaluating the actual preservation effect of nanoparticles. The antimicrobial activity of CEO-loaded nanoparticles against two dominant aquatic product spoilage bacteria, *Shewanella putrefaciens* and *Pseudomonas fluorescens* was evaluated. As shown in Figure 8, at all tested CEO concentrations (2%, 4%, and 8%), the SA-SCNPs exhibited stronger antibacterial activity than the SCNPs, with the inhibitory effect demonstrating a clear dose dependency. The enhanced antibacterial performance of SA-SCNPs was mainly attributed to the hydrophilic and strongly negatively charged shell formed on their surface. This shell significantly improved the colloidal stability and dispersibility of the nanoparticles through electrostatic repulsion, preventing aggregation and sedimentation, which allowed more active particles to diffuse effectively [88]. Meanwhile, this shell likely optimized the contact mode between the particles and bacteria via interactions such as hydrogen bonding and provided a potential responsive release mechanism, enabling the efficient release of antimicrobial components near the site of action.

The modified system’s excellent loading capacity and linear release profile were corroborated by a significant, concentration-dependent enlargement of the inhibition zones observed in the experiment. Its stable structure could carry a higher drug load without compromising dispersibility. The findings of this study are consistent with those Gholamhossein Tabar Valookolaei et al. [89], both discovering that SA encapsulation can significantly enhance the antibacterial activity of plant essential oil.

In summary, SA modification endowed SNPs with stable, hydrophilic, and negatively charged surface properties, greatly improving their delivery efficiency and controlled release behavior as carriers for hydrophobic essential oils. Consequently, they exhibited significantly enhanced and dose-dependent antibacterial activity in vitro. The targeted antibacterial activity of SA-SCNPs against *Shewanella putrefaciens* and *Pseudomonas fluorescens*—two dominant spoilage bacteria responsible for off-odors and texture deterioration in fish—highlights its direct application potential. Combined with its pH-responsive release, a potential application mode on real fish fillets or mince can be envisioned: as these bacteria proliferate in the later storage stages and elevate the local pH microenvironment, SA-SCNPs would release a high concentration of cinnamaldehyde precisely at the bacterial colonization sites, achieving “targeted antimicrobial action.” This could enhance preservative efficiency, potentially reduce the total amount of antimicrobial required compared to non-responsive systems, and may slow the development of bacterial resistance. Future work should focus on incorporating SA-SCNPs into edible coatings or active packaging films to validate their efficacy in inhibiting microbial growth, reducing total volatile basic nitrogen (TVB-N), and maintaining sensory qualities in real seafood during storage.

## 4. Conclusions

This study successfully developed a pH-responsive nano-delivery system by modifying SNPs with SA for encapsulating and controlling the release of CEO. Systematic screening identified 0.1% (*w*/*v*) SA as the optimal modifier, significantly improving SNP stability and monodispersity. The resulting SA-SCNPs exhibited a small particle size, high negative zeta potential, and up to 90% CEO encapsulation efficiency. FT-IR analysis confirmed core–shell formation via ionic and hydrogen bonding. SA-SCNPs showed intelligent pH-responsive release, with markedly higher cumulative release at an alkaline pH (7.0) simulating fish spoilage. Release kinetics followed the Ritger–Peppas model, indicating diffusion and matrix erosion mechanisms. In contrast, CS-SCNPs performed poorly due to alkaline aggregation. SA-SCNPs also demonstrated excellent stability during 28-day storage, under high ionic strength (≤300 mmol/L NaCl), and across temperatures (−18 to 25 °C). The enhanced antibacterial activity against *Shewanella putrefaciens* and *Pseudomonas fluorescens* is attributed to robust stability and pH-triggered on-demand CEO release. In summary, this work provides not only an intelligent preservation strategy for aquatic products but also a rational design principle—through comparative polyelectrolyte analysis and holistic performance optimization—for developing advanced, application-tailored delivery systems.

## Data Availability

The original contributions presented in this study are included in the article/Supplementary Material. Further inquiries can be directed to the corresponding author.

## Supplementary Materials

The following supporting information can be downloaded at: https://www.mdpi.com/article/10.3390/foods15071237/s1, Figure S1: Appearance of nanoparticles at different pH. SA (A), CS (B), Gel (C).
