# Supplementary material for: pH-TriggeredRelease of Cinnamon Essential Oil from Sodium Alginate-Shellac Nanoparticles: Rational Design, Enhanced Stability and Antibacterial Efficacy
Source: Foods. 2026 Apr 4;15(7):1237. doi: 10.3390/foods15071237 (PMC13074001; doi:10.3390/foods15071237)
Supplement: Supplementary file 1 [file foods-15-01237-s001.zip › foods-4208361-supplementary.pdf]

Supplementary material

pH-TriggeredRelease of Cinnamon Essential Oil from Sodium Alginate-Shellac Nanoparticles: Rational Design, Enhanced Stability and Antibacterial Efficacy

Sijing Liang [1], Ouyang Zheng [1,2], Jing Xie [3], Shucheng Liu [1,2] and Qinxiu Sun [1,2,*]

[1] College of Food Science and Technology, Guangdong Ocean University, Guangdong Provincial Key Laboratory of Aquatic Product Processing and Safety, Guangdong Province Engineering Laboratory for Marine Biological Products, Guangdong Provincial Engineering Technology Research Center of Seafood, Key Laboratory of Advanced Processing of Aquatic Product of Guangdong Higher Education Institution, Zhanjiang 524088, China; liangsijing0912@163.com (S.L.); zhengouyang07@163.com (O.Z.); lsc771017@163.com (S.L.)

[2] Collaborative Innovation Center of Seafood Deep Processing, Dalian Polytechnic University, Dalian 116034, China

[3] College of Food Science and Technology, Shanghai Ocean University, Shanghai 201306, China; jxie@shou.edu.cn

* Correspondence: sunqinxiugo@163.com

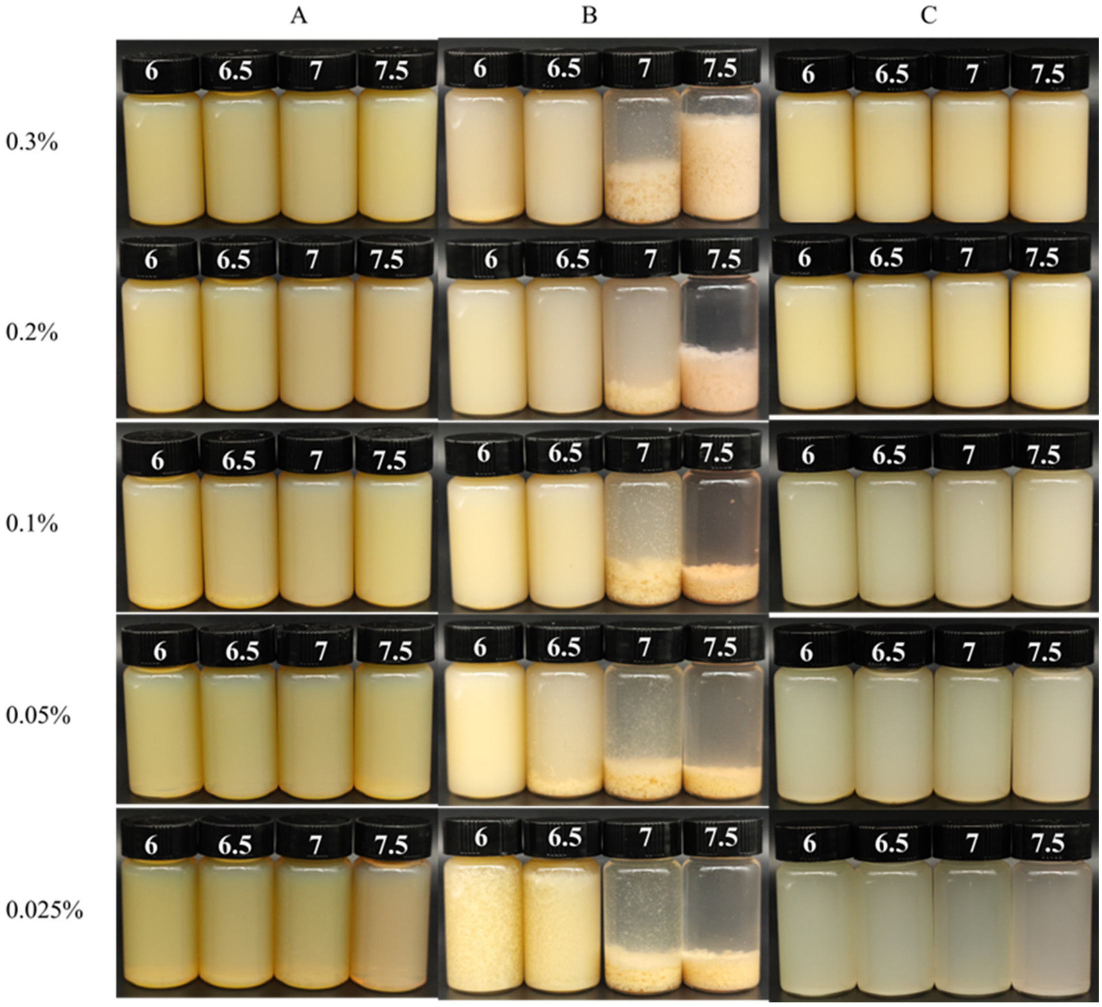

Figure S1. Appearance of nanoparticles at different pH. SA (A), CS (B), Gel (C).
